# The i-view® Video Laryngoscope Compared With the Macintosh Laryngoscope Does Not Enhance the Endotracheal Intubation Skills of Dental Students

**DOI:** 10.7759/cureus.66400

**Published:** 2024-08-07

**Authors:** Marina Takata, Mika Nishikawa, Satoru Eguchi, Kaori Takata, Hiroyuki Kinoshita, Shinji Kawahito

**Affiliations:** 1 Department of Dental Anesthesiology, Tokushima University Hospital, Tokushima, JPN; 2 Department of Anesthesiology and Intensive Care, Hamamatsu University School of Medicine, Hamamatsu, JPN

**Keywords:** simulation, manikin, video laryngoscope, macintosh laryngoscope, endotracheal intubation, dental student

## Abstract

Background

A disposable i-view^®^ video laryngoscope (Intersurgical Limited, Berkshire, United Kingdom) is yet to be used to educate medical and dental students, who must learn endotracheal intubation skills. Additionally, the advantage of the i-view^®^ use for the purpose, compared with the Macintosh laryngoscope, is unknown. We aimed to first determine whether the i-view^®^ video laryngoscope enhances endotracheal intubation skills among dental students compared with the Macintosh laryngoscope.

Methodology

A prospective, observational, simulation study was conducted among 67 dental students in their sixth clinical year of education. Intubation skills were evaluated on a computer-assisted simulator with a standardized manikin. Each student was asked to intubate using the conventional Macintosh laryngoscope and the i-view^®^ video laryngoscope in the trachea of the simulator’s manikin. We collected objective data, including the retroflection angle of the manikin, the maxillary incisor contact pressure, time from picking up the laryngoscope to ventilation, intubation success, and intubation delay. Each student was further asked to grade their subjective evaluation concerning the visual field, Cormack and Lehane classification, operability, stability, needed force for intubation, and easiness during intubation.

Results

Enrolled dental students quoted that the i-view^®^ video laryngoscope demonstrated better visual field, Cormack and Lehane classification, operability, and stability than the Macintosh laryngoscope. However, they felt intubation easiness could have been better using Macintosh than i-view^®^. Intubation time, failure rate, and delay rate did not differ between the two laryngoscopes. Nevertheless, the maxillary incisor contact pressure (median interquartile range (IQR)) during the intubation increased in the i-view^®^ intubation compared with Macintosh (32 (24 to 41) vs. 25 (18 to 35) N, p = 0.010).

Conclusions

We first demonstrated that the i-view^®^ video laryngoscope compared with the Macintosh laryngoscope does not enhance the endotracheal intubation skills of dental students. However, the possible repeated use as an educational simulator training tool may add some advantages to the experience of video laryngoscope in both medical and dental students.

## Introduction

The COVID-19 pandemic necessitated changes in the safety protocols of endotracheal intubation at every level of care. A video laryngoscope lets the operator stay further from the patient’s airway than conventional laryngoscopy. Thus, clinicians favor its employment to mitigate the risk associated with close patient contact [1]. Since then, a disposable i-view® video laryngoscope (Intersurgical Limited, Berkshire, United Kingdom) has become available in clinical practice [2].

Previous manikin studies with anesthesiologists demonstrated no significant difference in the intubation time between the Macintosh and the i-view® video laryngoscope without a significant difference in the intubation success rate [3,4]. A study with emergency medicine residents and attending physicians documented that the i-view® video laryngoscope has potential for the clinical setting, with all achieving rapid first-pass success for intubation [5]. These studies indicate that the disposable i-view® video laryngoscope has at least comparable performance for physicians upon orotracheal intubation with the conventional Macintosh direct laryngoscope.

Some studies suggest that at least 50 endotracheal intubations using a conventional direct laryngoscope are necessary to achieve a 90% success rate in patients [6]. Therefore, compared with the Macintosh laryngoscope, the video laryngoscope may easily exert endotracheal intubation skill achievement in personnel without clinical experience. The i-view® video laryngoscope is yet to be used to educate medical and dental students, who must learn endotracheal intubation skills. Additionally, the advantage of the i-view® use for the purpose, compared with the Macintosh laryngoscope, is unknown.

Therefore, we aimed to determine whether the i-view® video laryngoscope enhances endotracheal intubation skills compared with the Macintosh laryngoscope among dental students as learning endotracheal intubation using a video laryngoscope possibly improves the intubation skills of medical students [7]. We employed a computer-assisted simulator equipped with a standardized manikin for the purpose. The primary outcome of this study was whether the i-view® video laryngoscope decreases the intubation time using our simulator by dental students. The secondary outcome was if the i-view® reduces the maxillary incisor contact pressure.

## Materials and methods

The study was performed from November 2022 to October 2023 at the Department of Dental Anesthesiology, Tokushima University Hospital, Tokushima, Japan. Ethical approval for this study was provided by the Clinical Research Review Board of Tokushima University, Tokushima, Japan on May 30, 2022 (approval number: jRCTs062220047). This study was registered in the University Hospital Medical Information Network Clinical Trials Registry (UMIN000053465). Written informed consent was obtained from all dental students before their enrolment in the study. The procedures in the current study followed the Declaration of Helsinki and the ethical standards of the responsible committee on human experimentation. Dental students were in their sixth clinical year of education and had previously received theoretical lessons concerning the practice of anesthesiology but had no previous experience with intubation.

Study design

**Figure 1:**
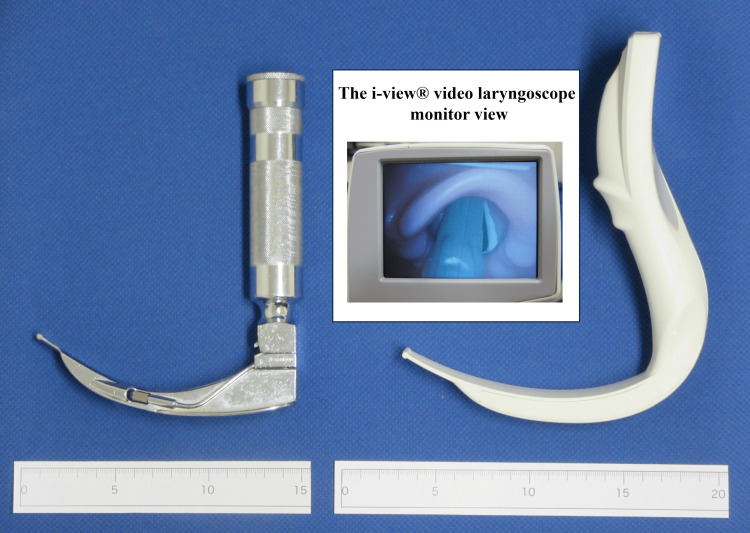
Pictures of the i-view® video (right) and the conventional Macintosh (left) laryngoscopes. Pictures of the i-view® video (right) and the conventional Macintosh (left) laryngoscopes employed in this study and a laryngeal view using the i-view® are shown. Please note that the i-view® blade is longer than the Macintosh’s and that the i-view® handle is thicker and more angulated forward than the Macintosh’s.

Intubation skills were evaluated on a computer-assisted simulator (Airway Management Simulator, Nihon Light Service, Inc., Tokyo, Japan) with a standardized manikin. The simulator allows real-time examination regarding quantitative analysis of airway maintenance, jaw-lift, sniffing position, incisor injury and ventilation, and time measurement during each manipulation using the built-in sensors within the manikin. Each student was asked to intubate using a conventional size-4 Macintosh and the i-view® video laryngoscopes the trachea of the simulator’s manikin (Figure 1). We chose a conventional size-4 Macintosh blade as it is the largest blade used in our dental anesthesia practice. Four trained dental anesthesiologists, including MT MN, SE, and KT, instructed how to use a Macintosh laryngoscope and i-view® video laryngoscope and explained the following measurements for 10 minutes. We collected objective data, including the retroflection angle of manikin (degree), the maxillary incisor contact pressure (N), time from picking up the laryngoscope to ventilation (intubation time, seconds), intubation success (intubation time <120 seconds, yes/no), and intubation delay (intubation time <60 seconds, yes/no). Successful endotracheal intubation was determined using the simulator with a built-in computer. Unilateral intubation of one mainstem bronchus was considered successful in this study. Each student was further asked to grade their subjective evaluation in relation to the visual field, Cormack and Lehane classification [8], operability, stability, needed force for intubation, and intubation easiness during intubation using a numerical rating scale (1, worst to 10, best other than Cormack and Lehane classification, 1 to 4). The students were permitted one intubation attempt using each laryngoscope during their evaluation of the manikin in random order. The Department of Dental Anesthesiology, Tokushima University Hospital provided a sealed envelope with the assignment, allocated to each student, either the Macintosh first or the i-view® first for the randomization.

Sample size determination

We used G*power Version 3.1.9.6 (Heinrich-Heine-Universität Düsseldorf, Düsseldorf, Germany) for the power calculation. A previous similar study determined a required sample size of 48 in each laryngoscope based on an alpha error of 0.05 and a beta error of 0.1 [9]. Enrolled students increased to allow for potentially incomplete data collection. Indeed, 67 students per group had 98% power at a two-sided alpha level of 0.05, regarding the intubation time difference of 10 seconds in this study. Accordingly, the enrolment of dental students was considered adequate.

Statistical analysis

Statistical analyses were performed using SPSS Statistics version 27 (IBM Japan Inc., Tokyo, Japan). Data are shown as the median and interquartile range (IQR) or number (%), while Wilcoxon signed-rank and chi-square tests were performed for group comparisons, respectively. Differences were considered statistically significant at p-values <0.05.

## Results

Table 1 presents the objective data taken by the simulator during tracheal intubation. Retroflection angle (40 (36 to 44) vs. 40 (36 to 45) degree, p = 0.855), intubation time (47 (40 to 63) vs. 49 (40 to 59) seconds, p = 0.876), intubation failure rate (0 (0.0) vs. 1 (1.5), p = 0.315), and intubation delay rate (18 (26.9) vs. 15 (22.4), p = 0.547) did not differ between the i-view® video laryngoscope and the Macintosh laryngoscope. However, the maxillary incisor contact pressure during the intubation significantly increased in the i-view® intubation compared with Macintosh (32 (24 to 41) vs. 25 (18 to 35) N, p = 0.010). These objective data indicate that the i-view® use does not improve intubation easiness in dental students.

**Table 1:** Objective data during tracheal intubation (n = 67 each). Data are shown as the median and interquartile range (IQR) or number (%), while Wilcoxon signed-rank and chi-square tests were performed for group comparisons, respectively. Differences were considered statistically significant at p-values <0.05. The asterisk (*) symbol indicates significant p-values (p < 0.05).

Objective data	Macintosh	i-view	P-value
Retroflection angle (degree)	40 (36 to 44)	40 (36 to 45)	0.855
Incisor pressure (N)	25 (18 to 35)	32 (24 to 41)	0.010*
Intubation time (second)	47 (40 to 63)	49 (40 to 59)	0.876
Intubation failure	0 (0)	1 (1.5)	0.315
Intubation delay	18 (26.9)	15 (22.4)	0.547

Table 2 shows subjective data from dental students during tracheal intubation. They quoted that the i-view® video laryngoscope demonstrated better visual field (8 (6 to 10) vs. 6 (4 to 8), p < 0.001), Cormack and Lehane classification (2 (1 to 2) vs. 2 (2 to 2), p = 0.018), operability (8 (7 to 9) vs. 7 (5 to 8), p = 0.004), and stability (8 (7 to 9) vs. 7 (6 to 8), p = 0.019) than the Macintosh laryngoscope. However, enrolled students felt intubation easiness was worse upon using the i-view® than the Macintosh (5 (3 to 7) vs. 7 (4 to 8), p = 0.016), whereas the needed force for intubation was the same between the two laryngoscopes (7 (5 to 8) vs. 7 (5 to 8), p = 0.479). These objective data also indicate that the i-view® use does not improve intubation easiness in dental students despite a better visual field.

**Table 2:** Subjective data during tracheal intubation (n = 67 each). Data are shown as median (interquartile range), while Wilcoxon signed-rank and chi-square tests were performed for group comparisons, respectively. Differences were considered statistically significant at p-values <0.05. The asterisk (*) symbol indicates significant p-values (p < 0.05). C-L: Cormack and Lehane

Subjective data	Macintosh	i-view	P-value
Visual field	6 (4 to 8)	8 (6 to 10)	<0.001*
C-L classification (1 to 4)	2 (2 to 2)	2 (1 to 2)	0.018*
Operability	7 (5 to 8)	8 (7 to 9)	0.004*
Stability	7 (6 to 8)	8 (7 to 9)	0.019*
Needed force for intubation	7 (5 to 8)	7 (5 to 8)	0.479
Intubation easiness	7 (4 to 8)	5 (3 to 7)	0.016*

## Discussion

In this study, the objective data during tracheal intubation demonstrated that intubation time, intubation failure rate, and intubation delay rate did not differ between the i-view® video and Macintosh laryngoscopes in the same retroflection angle. Our results agree with previous manikin studies with anesthesiologists documenting no difference in the intubation time between the i-view® video and Macintosh laryngoscopes without a significant difference in the intubation success rate [3,4]. Further, a study with emergency medicine residents and attending physicians who mostly had real patient experience with both direct and video laryngoscopy demonstrated that the i-view® has strong potential use for the clinical setting with all achieving rapid first-pass success for intubation [5]. Therefore, the i-view® video and the conventional Macintosh laryngoscopes are capable of supporting similarly for orotracheal intubation performance among personnel with and without tracheal intubation experience. However, in the present study, the maxillary incisor contact pressure during the intubation significantly increased in the i-view® intubation compared with Macintosh.

The maximum force applied on maxillary incisors was higher with the use of the i-view than with the Macintosh laryngoscope (i-view®, 32 N (24- 41 N); Macintosh, 25 N (18-35 N)). The maximum force applied on maxillary incisors was higher with the use of the video laryngoscope than with the Macintosh laryngoscope [10,11]. Another previous study demonstrated that the complete arch of the incisors has a maximum bite force ranging from 150 to 200 N [12]. Therefore, if the teeth are healthy, a peak force on the maxillary incisors of more than 150 to 200 N might be a risk factor for dental injury during laryngoscopy. Nevertheless, if the patient has preexisting dental problems, such as dental caries, periodontal disease, or abnormally positioned teeth, dental injury can be caused more easily [13] with much less lifting force. Actually, “minimal” force applied on the oral structures to prevent endotracheal intubation-related complications has not been well defined [14].

As shown in the pictures of the i-view® video laryngoscope and the conventional Macintosh laryngoscope in Figure 1, the i-view® blade is longer than the Macintosh’s. Moreover, the i-view® handle is thicker and more angulated forward than Macintosh’s. These structural differences must affect the augmented maxillary incisor contact pressure upon using the i-view®.

In this study, dental students mentioned that the i-view® video laryngoscope showed better visual field, Cormack and Lehane classification, operability, and stability than the Macintosh laryngoscope. Our results agree with a recent study documenting that the i-view® exhibited superior Cormack and Lehane grades and lower subjective difficulty scale scores than the Macintosh [3]. In novice intubators, shorter blades performed better, suggesting that intubation outcomes for novice trainees may be influenced by the size of the blade used [15]. However, longer blades tend to have better maneuverability, less pressure on the oral cavity, and more stress on the epiglottis [10]. However, the above subjective data were quite similar between the two studied laryngoscopes, although most data reached statistically significant differences. Therefore, the usefulness of the Macintosh and i-view® laryngoscopes may not differ much for the novice practitioner.

Excess force on the upper airway during laryngoscopy also increases sympathetic activities [16,17], causing adverse hemodynamic alterations such as hypertension, tachycardia, arrhythmia, and even cardiac arrest [18-21]. Therefore, endotracheal intubation should be performed with minimal force applied to the upper airway to prevent these consequences.

Enrolled students felt intubation easiness was worse upon using i-view® than Macintosh, whereas the needed force for intubation was the same between the two laryngoscopes. Video laryngoscopes minimize neck flexion or extension, and despite a clear view on the video screen, it can be difficult to direct the tube toward the glottis. It is thought that the shape of the stylet, which is an auxiliary device, may have an effect when operating the tracheal tube. This study compared i-view® and Macintosh laryngoscopes. As a result, Cormack and Lehane improved, but incisor pressure did not decrease significantly. Therefore, the conditions of use of the intubation aid may have influenced the results [22].

The results appear to be due to structural differences between the two laryngoscopes, as we mentioned above. These findings collectively suggest that the force applied to oral structures can be quantified as a marker of endotracheal intubation skill proficiency. As previously noted, in dental students, evaluation of the applied forces on oral structures during endotracheal intubation attempts using a simulator may help monitor the endotracheal intubation skill development among novices. The use of a simulation-based assessment system is harmless and repeatable [23].

Collectively, with objective data in this study, the i-view ® video laryngoscope possesses a particular difficulty in handling the scope during tracheal intubation, whereas the view appears better than the Macintosh direct laryngoscope. However, we do not suppose our study indicates that the i-view video laryngoscope is inferior in intubation training for dental students.

This study has a few limitations. First, in the current study, we employed the i-view® video laryngoscope only to improve the intubation skills of dental students. However, it is critical to note that our observation was made in a novice population. Second, when skilled anesthesiologists use the i-view®, it would provide better intubation conditions compared with the Macintosh. However, we do not have a clear answer to why the i-view® could not provide a better view. Moreover, video laryngoscopes, which are currently available in clinical practice, possess different structures and systems, indicating exertion of variable performance upon tracheal intubation [9]. The performance changes according to the patients’ factors related to intubation difficulty, for example, obesity [24]. Third, more importantly, novice practitioners may find it more challenging to guide the endotracheal tube through a two-dimensional video image than under a direct visual field. To overcome the skill bias, included students might be required to perform intubation maneuvers before and after the training, and after that, we may have to compare the changes in intubation success rates and other parameters in future studies. Additionally, using different types of video laryngoscopes and direct-view laryngoscopes requires different techniques [25]. Therefore, further studies with comparison among some video laryngoscopes and the Macintosh laryngoscope using a simulator with different clinical scenarios are required to explore the best intubation tool combination for students’ education.

## Conclusions

We first demonstrated that the i-view® video laryngoscope does not enhance the endotracheal intubation skills of dental students compared to the Macintosh laryngoscope. In contrast, the possible repeated use of the educational simulator training may add some advantages to medical and dental students’ experience of video laryngoscope use. We must remember that intubation techniques should be applied to patients after sufficient training. Further, we are not sure how the current results can be implied upon medical students.
